# A complementary approach for neocortical cytoarchitecture inspection with cellular resolution imaging at whole brain scale

**DOI:** 10.3389/fnana.2024.1388084

**Published:** 2024-05-23

**Authors:** Zhixiang Liu, Zhao Feng, Guangcai Liu, Anan Li, Hui Gong, Xiaoquan Yang, Xiangning Li

**Affiliations:** ^1^Britton Chance Center for Biomedical Photonics, Wuhan National Laboratory for Optoelectronics, MoE Key Laboratory for Biomedical Photonics, Huazhong University of Science and Technology, Wuhan, China; ^2^Key Laboratory of Biomedical Engineering of Hainan Province, School of Biomedical Engineering, Hainan University, Haikou, China; ^3^Research Unit of Multimodal Cross Scale Neural Signal Detection and Imaging, Chinese Academy of Medical Sciences, HUST-Suzhou Institute for Brainsmatics, JITRI, Suzhou, China

**Keywords:** cytoarchitecture, cortical parcellation, whole brain, macaque, 3D

## Abstract

Cytoarchitecture, the organization of cells within organs and tissues, serves as a crucial anatomical foundation for the delineation of various regions. It enables the segmentation of the cortex into distinct areas with unique structural and functional characteristics. While traditional 2D atlases have focused on cytoarchitectonic mapping of cortical regions through individual sections, the intricate cortical gyri and sulci demands a 3D perspective for unambiguous interpretation. In this study, we employed fluorescent micro-optical sectioning tomography to acquire architectural datasets of the entire macaque brain at a resolution of 0.65 μm × 0.65 μm × 3 μm. With these volumetric data, the cortical laminar textures were remarkably presented in appropriate view planes. Additionally, we established a stereo coordinate system to represent the cytoarchitectonic information as surface-based tomograms. Utilizing these cytoarchitectonic features, we were able to three-dimensionally parcel the macaque cortex into multiple regions exhibiting contrasting architectural patterns. The whole-brain analysis was also conducted on mice that clearly revealed the presence of barrel cortex and reflected biological reasonability of this method. Leveraging these high-resolution continuous datasets, our method offers a robust tool for exploring the organizational logic and pathological mechanisms of the brain’s 3D anatomical structure.

## Introduction

The cerebral cortex, a sheet-like formation on the outside of cerebrum, harbors approximately 16 billion neurons in humans (Van Essen et al., 2016). Given its pivotal role in cognitive function, structural and functional studies of cortex are paramount, particularly in humans and non-human primates. Since the pioneering work of Brodmann in 1900s, intensive efforts have been devoted to the delineation of cortical architecture. Regional variations in cell size, packing density and laminar organization within cortical transverse sections were considered as the proofs for cortical parcellation (Zilles and Amunts, 2010). Cytoarchitectonic differences among cortical regions not only reflect the anatomical features, but also the functional characteristics (Amunts and Zilles, 2015).

Several new techniques and reference standards were proposed for cortical parcellation, including anatomical circuit and functional connections. To elucidate the connectivity patterns of target cortical areas, the neurotracers were applied for parcellation based on the circuit uniqueness (Saleem et al., 2014; Borra et al., 2019). However, these methods based on two-dimensional (2D) representations proved less effective in a continuous three-dimensional (3D) space due to the limited information obtainable from the whole brain regions such as volumetric cortex (Hezel et al., 2012). With the invention of non-invasive stereo imaging such as MRI, it became feasible to visualize the whole brain structure and measure regional connectivity three-dimensionally with high-spatial resolution (Liu et al., 2018, 2020; Saleem et al., 2021). However, compared with optical imaging, the resolution of non-invasive stereo imaging remained a constraint, precluding the observation of finer structures. Consequently, observations often require verification using optical methods (Yan et al., 2022). Therefore, the cytoarchitecture observed by optical imaging still plays an irreplaceable role in brain structural analysis of and cortical parcellation.

More objective approaches had been proposed to quantify cytoarchitectonic feature on the cortex based on the optical imaging to overcome the subjective influence of the observer (Amunts et al., 2007). By these approaches, noteworthy results had been achieved (Amunts et al., 2020), in which the cortices were treated as ribbons on sections and the peaks of feature signal differences along the ribbons were set as the boundaries between cortical regions. However, the cortex was convoluted due to cortical expansion exceeding the surface area of subcortical nuclei and white matter (Van Essen et al., 2016), during which more cortical columns formed as the units for information processing (Molnár, 2011). This poses a challenge as a single section plane could not maintain the transverse plane of cortex at every position, especially in the brains of gyrencephalic animals like humans and macaques. To address this problem, an optimized blocking approach relied on MRI and surgical neuronavigation tools had been established (Novek et al., 2023) to obtain the ideal transverse view perpendicular to the sulcal orientation. However, it still required specialized equipment and expertise. Another strategy was relied on registration in which the slice images were registered and then stacked to form the volumetric data (Majka et al., 2021). While this strategy overcomes the limitations of 2D slices to some extent, the alignment process itself can introduce distortions to structural properties, such as cell distribution. Therefore, a more promising alternative is 3D optical imaging method, which possesses inherent self-alignment properties (Ragan et al., 2012; Gong et al., 2013; Seiriki et al., 2017). The abilities to delineate cytoarchitectonic image across the entire brain with subcellular resolution and 3D continuity (Xu et al., 2021; Zhou et al., 2022) had proven their potential for cortical analysis. Besides, how to extract cytoarchitectonic feature from large datasets remained a challenge, because cytoarchitecture encompassed both microscopic cellular morphology and mesoscopic architecture. Moreover, the convolution of cortex further compounded the complexity of the problem. Hence, an effective method for data analysis was needed.

In this study, we introduced a novel strategy for profiling cortical cytoarchitecture. The intact macaque brain was imaged using the fluorescence micro-optical sectioning tomography (fMOST) method, which enabled the revelation of neuron’s cytomorphological details with fluorescent dye (Hezel et al., 2012). Benefited from the cellular resolution in 3D, the cortical cells were classified based on their size, enhancing the contrast of the cytoarchitectonic differences across cortical laminates. The local densities of graded cells were extracted as cytoarchitectonic features and integrated into a surface-based framework which served as a representative of cortex. This strategy enabled us to detect significant differences in cytoarchitectonic feature signals across cortical regions, indicating its potential of for cortical architectonic profiling and parcellation.

## Materials and methods

### Animals

A 9-years old male *Macaca fascicularis* and 8-weeks old C57BL/6 J mice were used for this study. The monkey lived in individual cage under standard conditions (temperature 21 ± 2°C, humidity 60%), and were fed with food and water *ad libitum*. It was treated in accordance with the National Institutes of Health Guide for the Care and Use of Laboratory Animals. The experimental protocol and animal care protocols were approved by the Ethics Committee of the Kunming Institute of Zoology and the Kunming Primate Research Center (Approval No. IACUC18018). The mice were housed in normal cages in a specific-pathogen-free environment with a 12-h light/dark cycle, where the temperature and humidity were kept stable at 22–26°C and 40–70%, respectively. All mice had free access to food and water.

### Sample embedding and imaging

The macaque was anesthetized with sodium pentobarbital (45 mg/kg, intramuscular) and transcardially perfused with 4 L of 4°C 0.01 mol/L PBS and 1 L of 4% PFA. The mice were also deeply anesthetized with sodium pentobarbital (intraperitoneal) and subsequently perfused with 0.01 mol/L PBS, followed by 4% PFA. The brains were excised and post-fixed in 4% PFA at 4°C for 24 h. After fixation, each intact brain was rinsed overnight at 4°C in a 0.01 mol/L PBS solution.

The sample embedding and imaging of intact mouse brain and a right macaque hemiencephalon were followed the reported method (Zhou et al., 2022) (Supplementary Figure S1). In short, the post-fixed specimens were immersed in the solution of synthesized *N*-acryloyl glycinamide (NAGA) hydrogel monomer at 4°C for 12 h and 7 days, respectively and incubated at 40°C for 4 h for hydrogel polymerization. Then a home-made large-volume tissue imaging system was used for whole brain imaging. In the system, the samples were immersed in 2 mg/ml propidium iodide (P21493, Thermo Fisher, America) solution and the superficial sample was removed by a vibratome (VT1200S, Leica, Germany) after imaging in each “imaging-sectioning” cycle, so that the superficial sample could be stained in real time. A laser (Cobolt, Sweden) with wavelengths of 561 nm was used and the red emission signals were captured by a sCMOS camera (C13440-20CU, Hamamatsu, Japan). The imaging plane was set 10 microns below the sample section to avoid the effects of tissue loss or deformation caused by cutting, and the samples were imaged with a 10× objective (Olympus, Japan) four times every 12 μm thickness section, yielding the image volumes with 3D resolutions of 0.65 μm × 0.65 μm × 3 μm. The raw image stripes with overlap were stitched together implemented using C++ and the illumination intensities were corrected with polynomial curves as the preprocessing. The code is available at http://atlas.brainsmatics.org/a/zhong2019 (Zhong et al., 2021).

For the comparison of image processing strategy between 2D and 3D, the embedded macaque brain was sliced with vibrating microtome to get a 100-μm thick coronal plane section. The brain slice was stained with propidium iodide for 0.5 h and then imaged with laser scanning confocal microscope (Leica DMi8, Germany). The 20x objective was used and the step size of z direction was 0.5 μm to get a cellular 3D resolution of 0.446 μm × 0.446 μm × 5 μm. And a strip area of cortex from outer surface to inner was selected for imaging to get an image stack with 2000 μm in radial length, 350 μm in span and 75 μm in thickness.

### Size-depend cell grading and image enhancement

The original images, with a resolution of 0.65 μm × 0.65 μm, were utilized as the input for cell size grading. Initially, a morphological top-hat filtering was applied to each image to suppress the background signal. This filtering used a structural element (SE) slightly larger than the largest cell body, with a radius of 20 pixels. Subsequently, the filtered images were binarized with local adaptive thresholds to segment the foreground patches and three rounds of morphological opening operations were performed, with increasing radii of disk-shape structural elements (6, 10, and 14 pixels). This stepwise elimination of small patches left only the larger cells. Finally, each foreground pixel was assigned a cell size grade.

Given that pixels were assigned three grades, the foreground pixels of each image were distributed across three image channels, corresponding to red, green, and blue, respectively. These images were termed “cell-graded images.” The same processing steps were applied to the entire image stack. To generate a composite view, adjacent cell-graded images were projected using the mean, converting the binary images into continuous-value images. Since the absolute value ranges differed significantly across the three channels, adaptive brightness adjustments were made to each channel. The resulting images were designated as “enhanced images.”

### Image processing strategy comparison

An image volume of macaque brain cortex, exhibiting cellular and isotropic resolution, was employed for this comparative analysis. The image volume was binarized slice by slice in three orthogonal planes. Following binarization, Gaussian filtering was applied to the results, and a subsequent binarization step was performed using a threshold of 0.5. In the following analysis, most steps remained the same except SE. In the previously introduced 2D method, disk-shaped SEs were utilized for morphological opening operations. However, for the 3D method, ball-shaped SEs were employed instead. Finally, each voxel in the image volume was assigned a grade number.

The number of voxels within each grade was counted along the cortex radial direction generating a signal curve. The pattern of this curve could be discerned based on the number and positions of peaks and valleys. If there were minimal systematic differences between the two methods, the curve patterns should exhibit similarity.

### Cortical segmentation

To minimize computing and memory consumption, the serial images were down-sampled to form an image volume an isotropic resolution of 12 μm × 12 μm × 12 μm. Then the image volume was automatically segmented using Brain Extraction Tool in FMRIB Software Library (FSL^1^)to obtain a preliminary segmentation. During this process, the voxels were classified into three categories: grey matter, white matter and imaging background.

Manual verification was then performed to correct any unexpected segmentation errors. Additionally, irrelevant subcortical regions and the cortical layer I were removed in this step. This was necessary because *in vitro* samples often exhibit closely spaced sulcal surfaces on both sides, which could interfere with subsequent surface extraction and modeling of the cortex.

For manual segmentation, a proprietary software tool was utilized. This tool allowed for efficient segmentation by automatically interpolating every local operation across a specified thickness interval. To further enhance accuracy, operations were executed in three orthogonal views, minimizing the impact of oblique section planes that could result in a blurry GM/WM interface.

Finally, the desired segmented cortical volume should form a contiguous entity with no topological holes or rings, ensuring a clean and accurate representation of the cortical structure.

### Establishment of cortical coordinate system

The cortical streamlines were generated by simulating the behavior of electric field lines within the cortical region. The interfaces between cortical layer I and II (outer surface) and between gray matter and white matter (inner surface) were defined. These interfacial voxels were assigned fixed potential values (0 for outside and 2000 for inside). The potential values of the remaining voxels within the cortex were calculated to create a continuous potential field in 3D space ensuring consistency with the fixed surfaces. The final potential field was determined by solving a Laplacian equation.

The 3D gradient direction of the potential aligned with the direction of the streamline at each voxel. By tracing these streamlines through the cortex, we obtained a representation of cortical thickness, measured by the length of the streamlines. The relative positions of voxels along these streamlines served as a measure of radial depth within the cortex. Additionally, we identified the midpoint voxels of each streamline to construct a mid-thickness surface. Each voxel on this surface was uniquely labeled with an ID number, serving as a lateral coordinate. The remaining voxels were labeled based on their connections to the mid-thickness surface voxels via the streamlines.

Using this coordinate system, we defined radial units by grouping together streamlines that were in close proximity based on the proximity of their mid-thickness surface voxels. The k-Nearest Neighbor (KNN) method was used here to identify the nearest mid-thickness surface voxels, with *k*-values set to 1,000 for the macaque brain and 50 for the mouse brain. As a result, each voxel on the mid-thickness surface was associated with a specific radial unit. It is noteworthy that adjacent radial units had overlapping regions, resulting in a series of continuous signals that captured the spatial variation across the cortex.

### Cytoarchitectonic signal counting and clustering

With the established coordinate system, the cortex was laterally divided into radial units, and these radial units were further stratified into multiple laminates based on their relative depth. Since the somas pixels in the raw images were already labeled with grade numbers, we calculated the foreground pixel ratio in each lamina of each radial unit as a measure of cell distribution density. Initially, this calculation was performed within each 12 × 12 × 12 cubic volume, and then the results were aggregated at the level of radial units. In this study, we defined 20 laminates and considered 3 cell size grades. Consequently, the cytoarchitectonic pattern of a single radial unit was represented by a 20-by-3 signal matrix, effectively a 60-dimensional vector.

To facilitate further analysis, the dimensionality of these vectors was reduced using principal component analysis (PCA). The scores of the principal components that explained 95% of the total variance were then normalized and used as the basis for clustering. Subsequently, the radial units were clustered using the K-means method, providing an initial state for Markov random field clustering.

### The surface-based cortical cytoarchitectonic tomogram and parcellation

The voxels located on the mid-thickness surface were designated as vertices to generate a patch surface. The color assigned to each voxel was based on the cytoarchitectonic signal intensity (in the case of tomographs) or the group number (for parcellation) of the corresponding radial units. For tomographs, a patch surface array was generated to visualize the cytoarchitectonic pattern. This array represented the pattern across nine equally divided intervals of relative depth, ranging from 5 to 95%, for each cell size grade.

## Results

During the investigation of the cortical cytoarchitecture features, several steps could be summarized. Cells were recognized and classified based on morphological features locally in the high-resolution image. Then the distributions of different types of cells were counted within a large span with less spatial resolution requirement. Finally, laminar concepts were generated and regional cytoarchitectonic variations were identified. In our study, this thought had been followed.

### The whole macaque hemisphere imaging datasets

**Figure 1:**
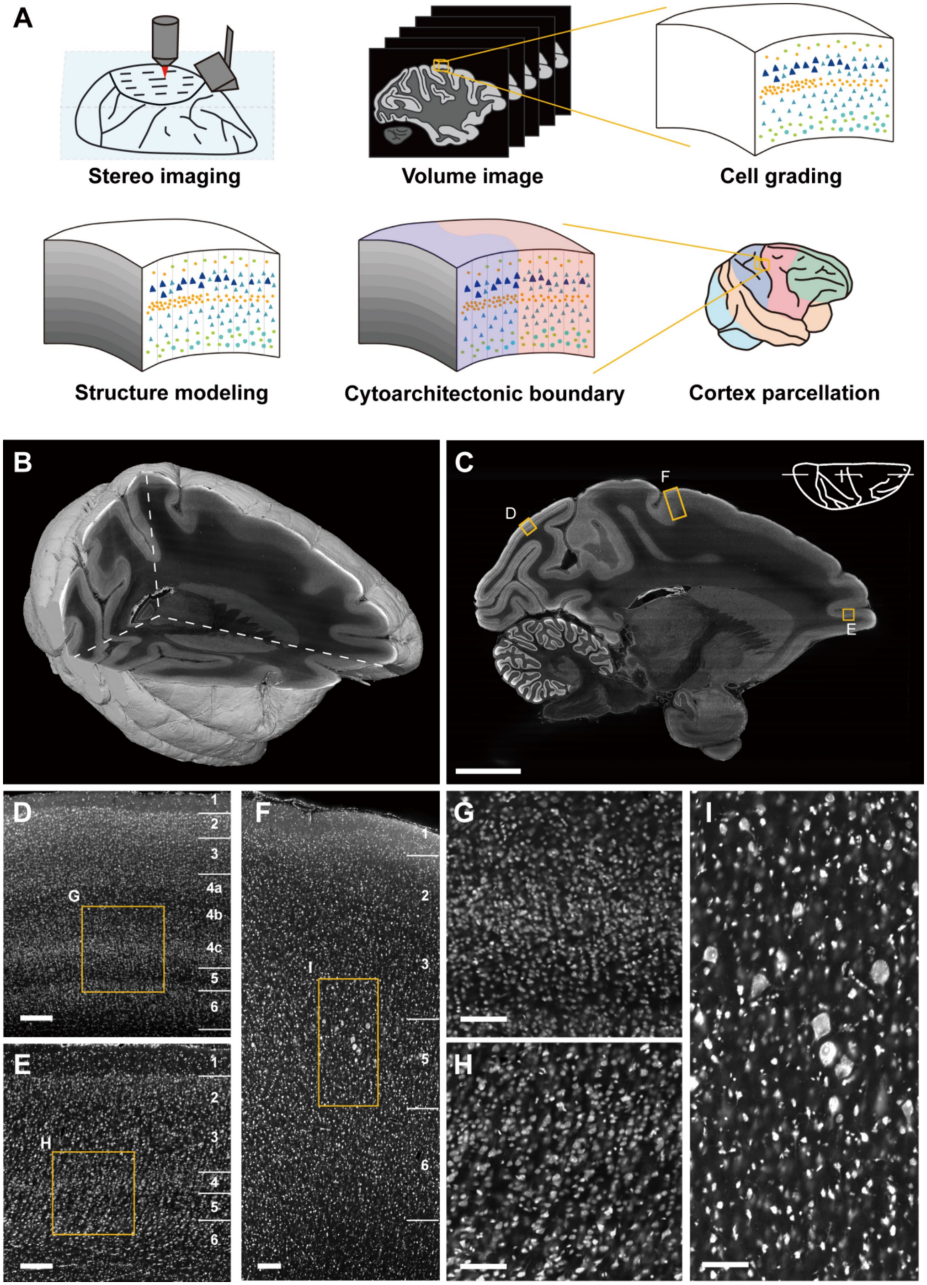
Cortical cytoarchitectonic profile with whole macaque hemisphere imaging datasets. **(A)** The overview of the pipeline. **(B)** The volume rendering and three orthogonal sections of imaging datasets. **(C)** A representative sagittal section. Scale bar, 10 mm. **(D–F)** The enlarged images from **(C)** shown typical cortical cytoarchitecture in V1, M1 and 46v, respectively. Scale bars, 200 μm. **(G–I)** The enlarged images from **(D–F)** presented the morphological details of cells. Scale bars, 100 μm.

Through the large-volume tissue imaging system (Zhou et al., 2022), we imaged the whole macaque hemisphere integrally and continuously in 3D with a resolution of 0.65 μm × 0.65 μm × 3 μm (Figures 1A,B and Supplementary Figure S2). Owing to the high-resolution on imaging section, the cortical cytoarchitectonic details were sufficiently captured (Figure 1C). The differentiation of cell packing density together with soma morphology highlighted the laminar cortical architecture. As shown, multiple complex laminates could be observed in primary visual cortex (V1) where the neurons were relatively small (Figures 1D,G). In the ventral bank of principal sulcus (46v), the typical granular layer in primate prefrontal cortex could be seen (Figures 1E,H). In primary motor cortex (M1, or agranular frontal area F1), the representative large pyramidal neurons could be found, with imperceptible laminar transitions (Figures 1F,I). The observed differences indicated the feasibility of cortical cytoarchitecture profiling in 3D space with these datasets, enabling image signal extraction and quantitative statistical analysis.

### The cortical cytoarchitectonic feature enhancement based on cell size grading

**Figure 2:**
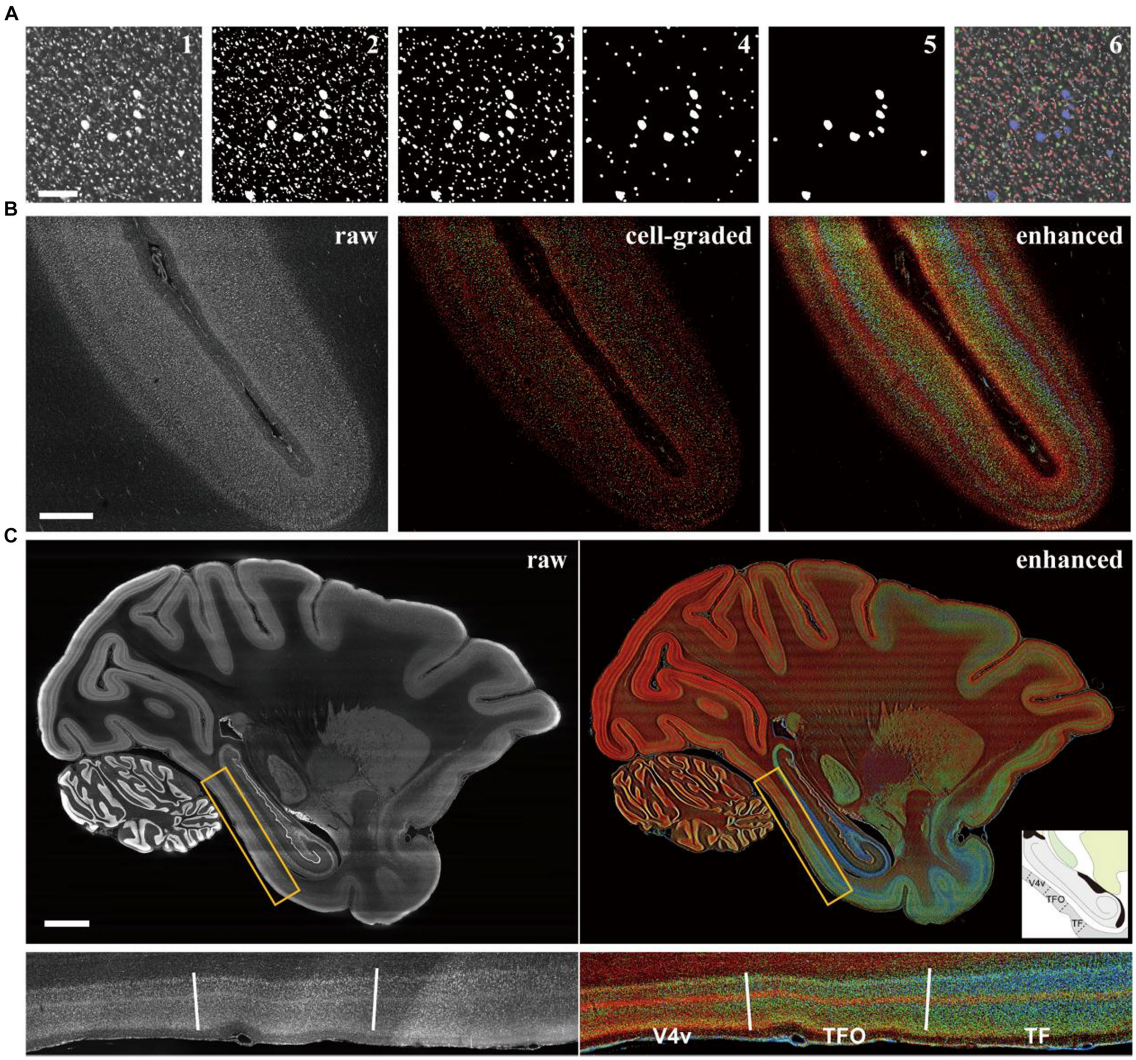
The cell grading manipulation enhance the cytoarchitectonic contrast between cortical laminates and regions. **(A)** The images were presented to explain the grading principle according to cell size with image morphological manipulation. The results after each step were shown in order: the raw (1), the binarized (2), the morphological opening filtered in three rounds (3–5), and the raw image labeled with colors of grade (6). Scale bars, 200 μm. **(B)** The comparison of the raw, the cell-graded and the enhanced image from the section of intraparietal sulcus (the definitions of images were in methods). Scale bars, 1 mm. **(C)** The comparison of the raw and enhanced images in whole brain level and a local area. The candidate boundaries were drawn with solid lines and the names of cortical regions were labeled: V4v, the ventral part of visual area 4; TFO, area TFO of the parahippocampal cortex; TF, area TF of the parahippocampal cortex. Scale bars, 5 mm.

Cell size is a pivotal aspect of cortical cytoarchitecture, and its distinction could greatly enhance laminar contras. Traditionally, evaluating cell size involved segmenting individual cells in images and measuring their 2D areas or 3D volumes. However, despite the challenging associated with isolating adhered cells (Costantini et al., 2021), the computational costs for processing billions of neurons across the entire macaque cortex (Collins, 2011) are immense. In this study, we employed traditional image morphological filtering for size grading. The images with original resolution were binarized using adaptive threshold to segment the cell signals as foreground. Subsequently, several rounds of morphological opening were applied to the binarized images to eliminate small signal patches (Figure 2A, III-V). The filtered images were then overlaid and the pixel values were accumulated to form grades (Figure 2A, VI). The results showed that large pyramidal neurons (labeled blue) in M1 were accurately distinguished from the neighboring smaller neurons (labeled red and green), demonstrating the effectiveness of our method in classifying cells based on size.

At the mesoscopic scale, the distributions of cells with different sizes manifests as laminar texture. However, the laminates may exhibit ambiguous boundaries in a single image (Figure 2B middle). In such cases, we observed that the laminar contrast was prominently enhanced in the mean projection of processed images (Figure 2B right) compared to the original images (Supplementary Figure S3A). The cell distribution densities were represented by brightness with different colors corresponding to three size grades. This allows for the presentation of more laminar details that are undetectable in unenhanced images. A quantitative analysis of laminar signal differences was also conducted, revealing that the enhanced images exhibited more pronounced fluctuations and additional peaks (marked by the arrows in Supplementary Figure S3B) indicating more detectable information. One consideration was that the area of soma section might be affected by the position and angle of section plane. Therefore, an evaluation was conducted to assess the difference between 2D morphological method and 3D. By employing ball-shaped structure elements instead of disk-shaped ones on an image volume of cortex with cellular 3D resolutions acquired through laser scanning confocal microscope, we found limited difference between the two methods. While there were slight variations in the absolute voxel counts, the overall trends of the curves were comparable (Supplementary Figure S4E). The same laminar pattern could be recognized using both methods. Given the computational considerations, the 2D-based method was ultimately utilized.

When this strategy was applied to the entire brain, cytoarchitectonic feature could be quickly observed on a large scale (Figure 2C), owing to the presentation of cortical laminar texture through brightness and color. Some cortical region boundaries were also identifiable where laminar patterns underwent significant changes and these boundaries were consistent with the existing atlas (Saleem and Logothetis, 2012). It is noteworthy that the shape of neuronal sections can vary when the cortex is sliced at different angle, especially for pyramidal cells, which could potentially affect the estimation of cell size. However, the laminar texture remained discernible even in areas where the cortex appeared curled in coronal and horizontal sections (Supplementary Figure S5), indicating that the effect of section angle was limited.

### The 3D framework of cortex structure

The two-dimensional (2D) result above had showed the potential of extracting cortical cytoarchitectonic features and performing regional parcellation based on enhanced images according to cell size grading. However, a problem persisted: when the cortices were parallel to the section plane, this led to ambiguous texture and a low utilization rate of the sample. Additionally, it was hard for observers to generate an idea of cortical parcellation in 3D space based on the observation of 2D image series. A more intuitive approach would be to visualize the cytoarchitectonic features on a surface (Van Essen et al., 1998). Therefore, a framework was established to simplify the cortex structure.

**Figure 3:**
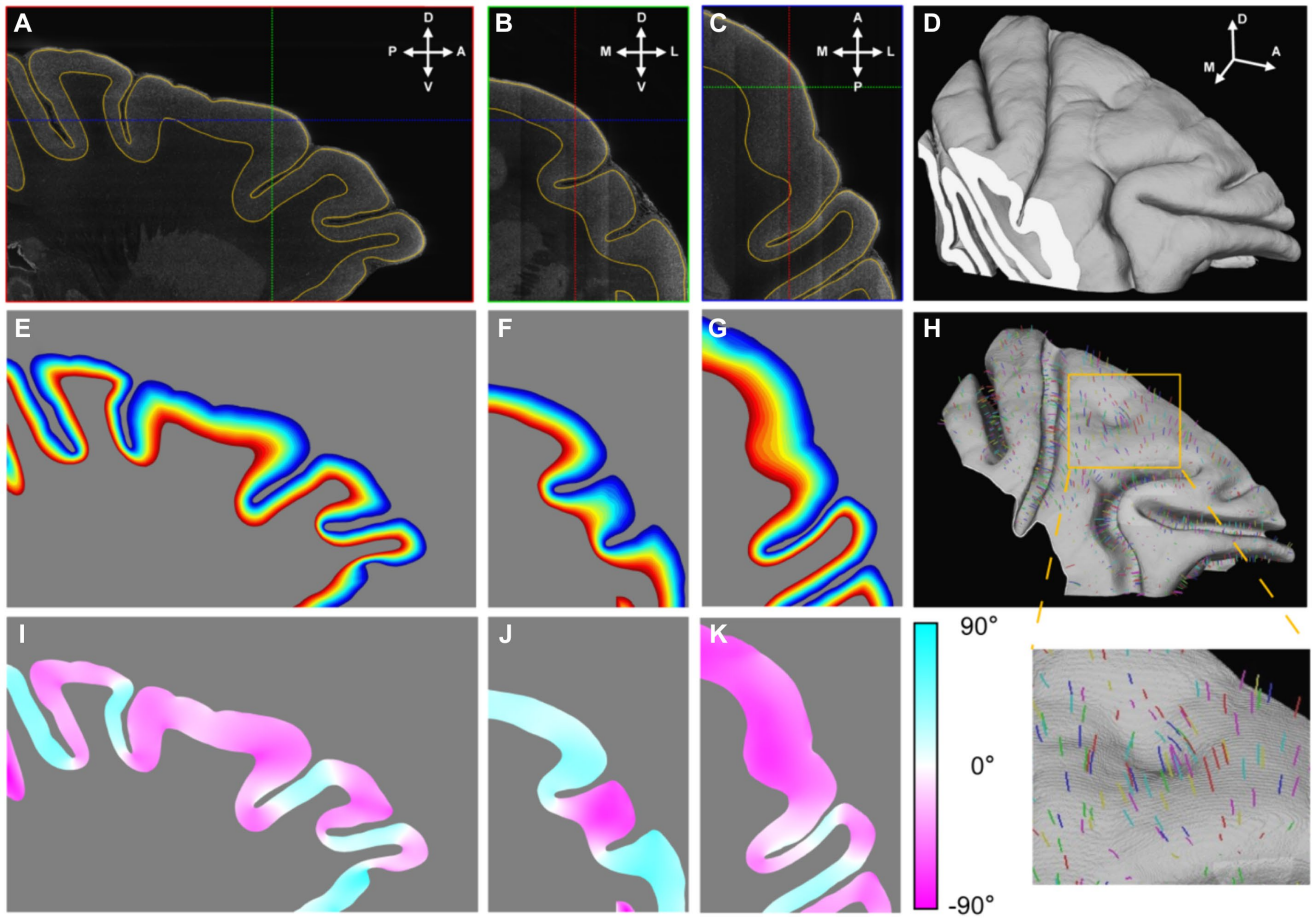
The establishment of cortical coordinate system. **(A–C)** Three orthogonal sections were presented in which the outlines of segmented cortex were drawn with yellow lines. The cross lines in each section indicated the positions of the other two sections. The anatomical directions were indicated with arrows: A, anterior; P, posterior; D, dorsal; V, ventral; M, medial; L, lateral. **(D)** The segmented cortex was presented by volume rendering. **(E–G)** The relative cortical depths were displayed with gradient color that the shallow laminates were displayed with cool colors and the deep with hot. **(H)** The mid-thickness surface was rendered and part of streamlines were drawn in random color. **(I–K)** The values of angles between the section planes and the streamline directions at their intersections were displayed with gradient color. The positive value meant the angle direction away from the observer.

The dorsolateral part of the frontal cortex was selected and segmented. Initially, a low-resolution image volume was pre-segmented using commonly utilized MRI software (FMRIB Software Library, FSL) to eliminate the background and white matter. Subsequently, the subcortical structure and cortical layer I were manually removed. With the help of an in-house labeling interface, the cortex was segmented continuously in 3D (Figures 3A–D). Notably, even in regions where the section planes were oblique relative to the cortices, resulting in indistinguishable grey matter (GM)/whiter matter (WM) interfaces in 2D images, the segmentation results remained equally precise.

Following binary segmentation, a cortex coordinate system was established. Given that cortical laminates are locally parallel and the cortical columns are perpendicular to these laminates, the cortex could be accurately modeled using a 3D Laplace’s equation. This modeling approach enabled the simulation of cortical laminates as an isosurface and the corresponding gradient direction of the cortical columns. Once the gradient directions were calculated, streamlines were traced from the interface between cortical layer I/II to the GM/WM interface, following the gradient direction. The distances along these streamlines were then normalized and were treated as relative cortical depth (Figures 3E–G). Based on this relative cortical depth, the mid-thickness surface was extracted as a simplified representative model of cortex structure (Figure 3H). The mid-thickness surface served as a lateral reference, as any point in cortical space could be mapped to a corresponding point on this surface. Combined with the relative depth information, the cortex coordinate system was established. This system allowed for the cortex to be divided into radial units, which served as the fundamental units for the surface-based analysis (Supplementary Figure S6). It is worth emphasizing that this 3D modeling approach was more precise than modeling based on 2D slices, because the streamlines cross the section plane in most time (Figures 3I–K) and it was consistent with the fact.

### The surface-based cortical cytoarchitectonic tomogram

After dividing the cortex into radial units, the distribution density was measured within radial units by calculating the percentages of foreground pixels, and the values were mapped onto the mid-thickness surface, providing a tomographic visualization of cytoarchitectonic signal.

**Figure 4:**
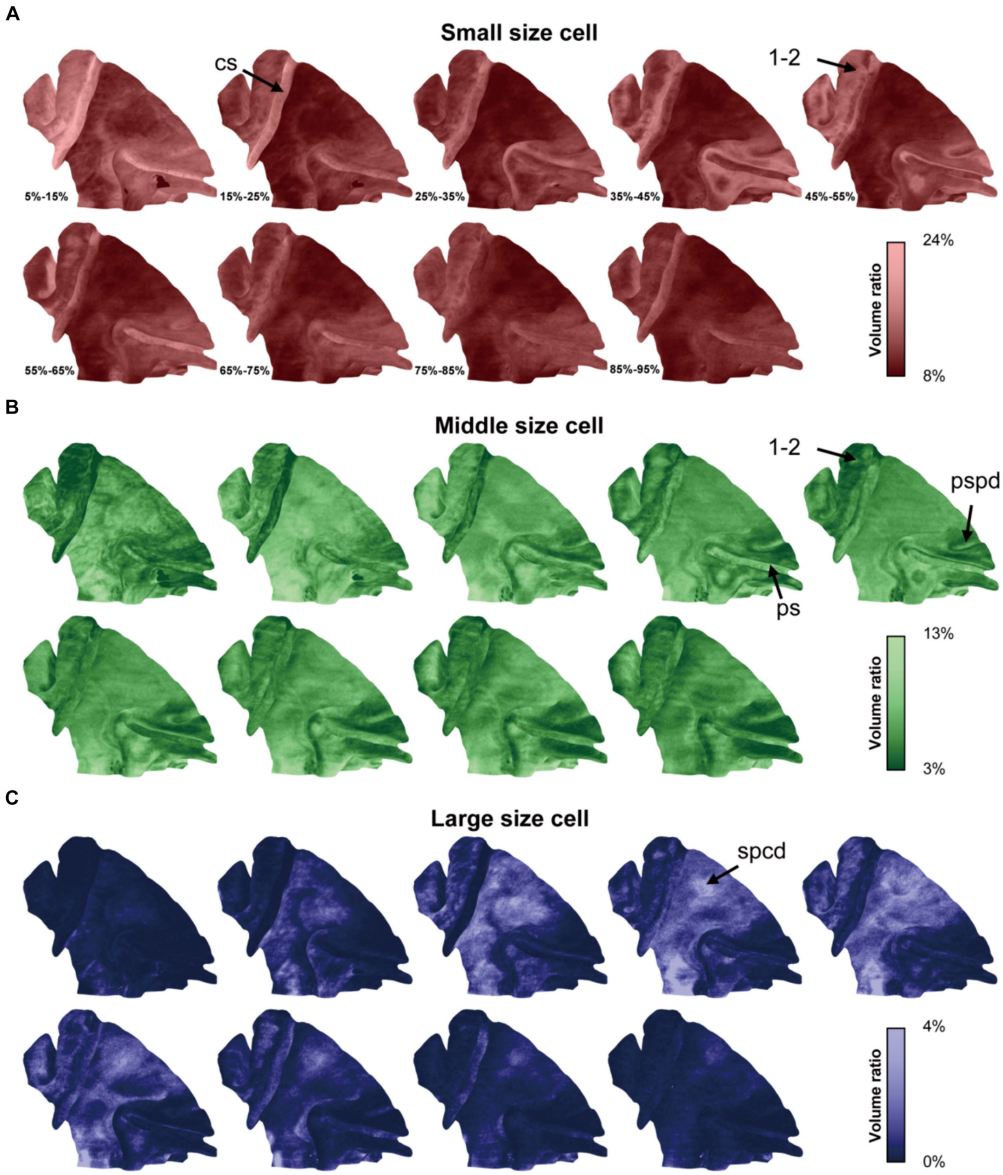
The cytoarchitectonic tomographic presentation of macaque cortex. **(A–C)** The panels were corresponded to three cell size grades and the cell distribution densities in different depth intervals from superficial to deep were projected on mid-thickness surface in each panel. The depth intervals were labeled in **(A)**. The arrows pointed to the sulci and region, including: cs, central sulcus; ps, principal sulcus; pspd, posterior supraprincipal dimple; spcd, superior precentral dimple; 1–2, somatosensory areas 1 and 2.

Utilizing this approach, the cytoarchitectonic pattern in each cortical laminate was expansively displayed across the lateral span (Figure 4). Generally, the ratios of cells of different sizes were consistent with the result obtained from the 2D-based analysis, while the laminar textures were also presented in a novel and distinct form. For example, a notable increase in the density of small cells was observed at the middle depth in somatosensory areas 1 and 2 (1–2, Figure 4A), while the opposite trend was evident for the cells of medium size (Figure 4B). This pattern is characteristic of the granular layer. Additionally, the cytoarchitectonic patterns exhibited uniformity along the sulcal contours but greater variability perpendicular to them. This phenomenon was evident in the central sulcus, the principal sulcus and even the posterior supraprincipal dimple (Figures 4A,B). Notably, even subtle anatomical features like the superior precentral dimple, which appears as a shallow indentation on the cortical surface, exhibited changes in cytoarchitectonic pattern. Specifically, a high percentage of large cells was observed at the middle depth, manifesting as a bright spot on the surface visualization (Figures 3D, 4C). This finding suggests that cytoarchitectonic patterns may reflect underlying cortical developmental trajectories.

### The radial units cluster based cortical parcellation

**Figure 5:**
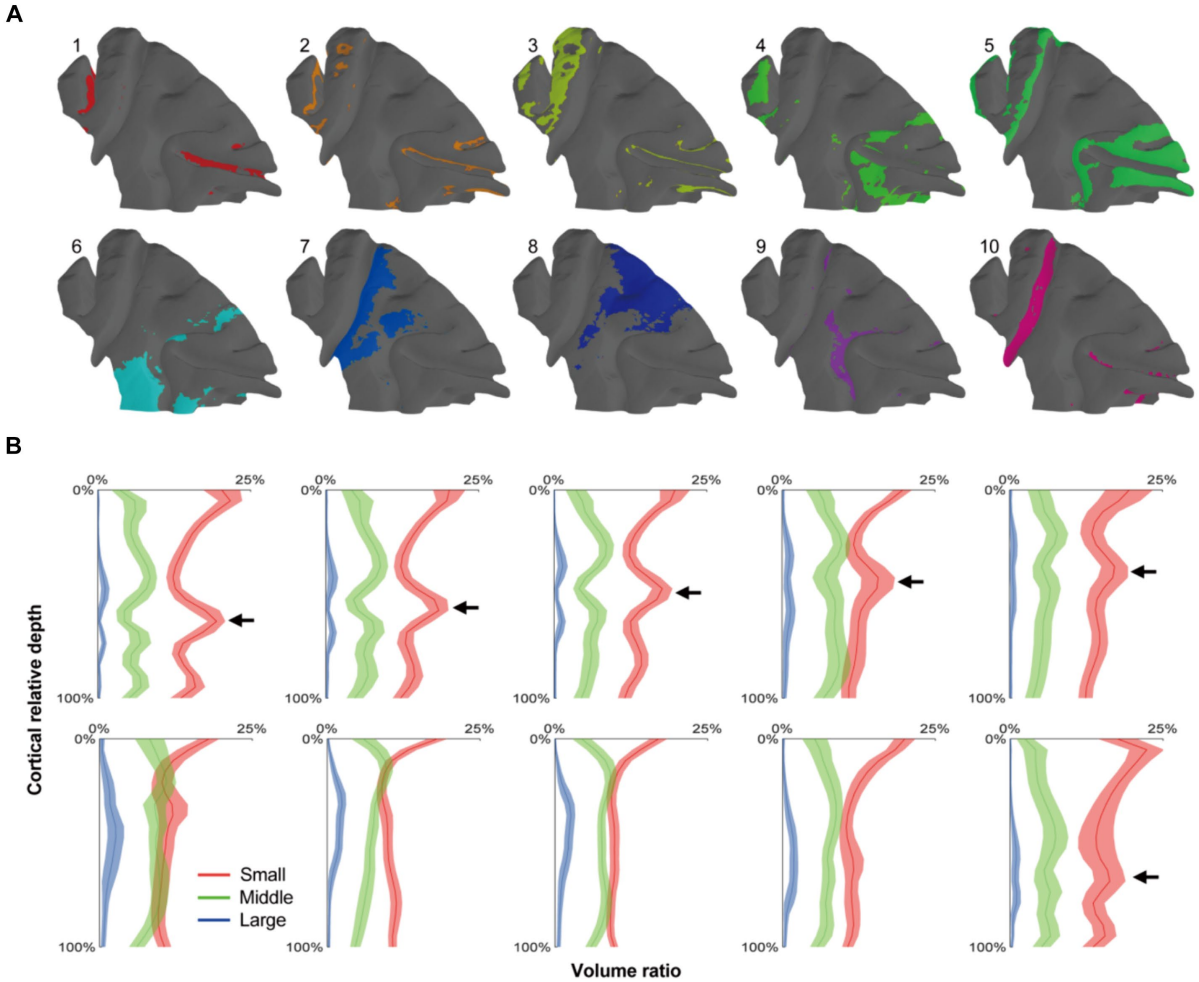
The cortical parcellation based on radial units cluster. **(A)** The radial units were clustered into 10 clusters, and each area were painted with colors on the mid-thickness surface. **(B)** The means of cell distribution densities for every units group were drawn as curves and the shaded areas represented the standard deviations. The arrows indicated the positions of granular layers which presented a gradient trend from cluster 1–5.

**Figure 6:**
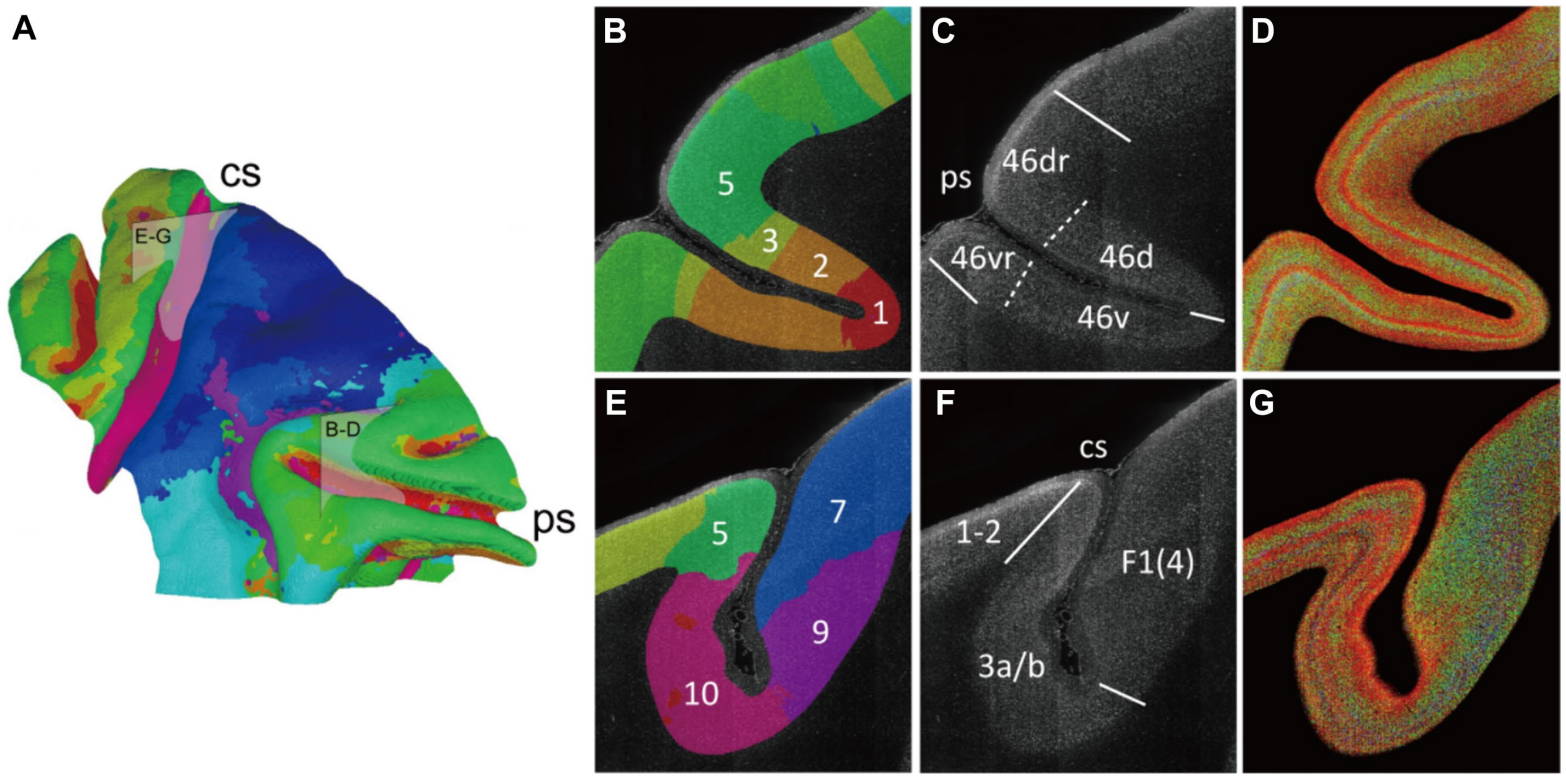
The varied cytoarchitectonic pattern transverse the sulcus. **(A)** The cluster result was shown on the mid-thickness surface as a summary of Figure 5A. And two boxes were drawn to indicate the section positions for **(B–G)**. **(B–G)** The principal sulcus which was mainly occupied by the area 46 and the central sulcus were focused. The brain slice was labeled with cluster color **(B,E)**. The border lines were manually drawn in accordance with the atlas and relevant reference. The boundaries in the reference atlas (Collins, 2011) were indicated with solid lines, and the extra boundaries in the reference article (Van Essen et al., 1998) were labeled with dotted line **(C,F)**. The enhanced 2D images were presented as cytoarchitectonic reference **(D,G)**. The abbreviation of the region name: 46d/v(r), the dorsal and ventral (crowns) of areas 46; 1–2, somatosensory areas 1 and 2; 3a/b, somatosensory areas 3a and 3b; F1(4), agranular frontal area F1 (or 4).

Following the progress above, the selected cortex was parceled by clustering the adjacent radial units together using Markov random field method, resulting in the designation of 10 distinct clusters (Figures 5A, 6A). Within the principal sulcus, a gradual shift in cytoarchitectonic patterns was observed across clusters 1, 2, 3, and 5. These clusters were spatially arranged from the internal to external part of sulcus (Figure 6B), exhibiting a consistent increase in the relative cortical depths of the granular layer (indicated by the arrows in Figure 5B). This finding underscores the cytoarchitecture pattern of area 46, in which the cytoarchitectonic differences between walls and crowns of principal sulcus were greater than those between dorsal and ventral regions (Figures 6B,D). Although previous studies have identified these distinctions (Cruz-Rizzolo et al., 2011), the potential parcellations had not been incorporated into the existing atlas (Saleem and Logothetis, 2012) (Figure 6C). Another notable change was detected in the central sulcus. Here, clusters 5, 10, 9 and 7 were arranged in order from posterior to anterior (Figure 6E). The granular layers initially shifted towards deeper laminate and then became undetectable, reflecting a dramatic cytoarchitectonic transition from somatosensory areas 1 and 2 through somatosensory area 3a/b, to primary motor cortex (Figures 6F,G). It is noteworthy that the cluster 10, corresponding to the somatosensory area 3a/b (Figures 6E,F), exhibited the most concentrated distribution and the most specific cytoarchitectonic pattern (Figures 5A,B), indicating the unique functional role of this area. Concurrently, a vast and relatively uniform area was occupied by clusters 7 and 8, displaying minimal intra-group variations (Figures 5A,B). However, subtle change could still be observed, such as variations in the number of medium-sized cells within deeper laminates. Overall, despite the fragmented nature of the results, they can still serve as useful references for manual cortical parcellation. It is also worth emphasizing that cytoarchitectonic similarities can exist across different cortical regions, providing insights into the organizational principles of the brain.

We also compared our cytoarchitecture-based cortical parcellation with the macaque brain atlas obtained from multimodal data (Collins, 2011) (Supplementary Figure S7). In the atlas, the area anterior to the central sulcus is divided into three subregions F1, F2, and F4 of agranular frontal area. While in our results, the corresponding area was occupied by clusters 7 and 8. Exactly, cluster 7 appeared as two isolated regions separated by cluster 8, which coincided with the partitioning pattern of the atlas. Meanwhile, although region F5 is also part of agranular frontal area according to its name, this region corresponded to cluster 6 in our results and exhibited a dense distribution of small-sized cells suspected to be the granular layer (Figure 5B). On the other hand, the high consistency of cytoarchitecture along the sulcal contours found in this study was also consistent with parcellation pattern in atlas (46d, 46v and 1–2). Although the subtle and gradual differences in cytoarchitecture among these regions proposed in our study are not reflected in the atlas, this phenomenon could still reflect the differences within the regions.

### The methodological verification

To assess the biological plausibility and methodological universality of this approach, similar operations were performed on mouse brain specimen (Supplementary Figure S8). Several boundaries between regions could be clearly delineated, reflecting regional characteristic (Supplementary Figures S8B,C). Notably, the typical texture of barrel cortex was observed in the barrel field of the primary somatosensory area (SSp-bfd, Supplementary Figure S8B), where each barrel appeared as a dark spot. In the radial units clustering analysis (Supplementary Figure S8D), the parcellation pattern closely resembled previously reported findings (Wang et al., 2020). These cytoarchitectonic tomographic presentations were biologically significant and not merely artifacts of the analysis.

We also investigated the impact of radial unit size. When using smaller radial units, the images were sharper and contained more granules. Conversely, with larger radial units, the granules appeared smoother. Nevertheless, despite changes in image granularity or smoothness, the overall signal pattern in the macaque cortex remained relatively unaffected (Supplementary Figure S9A). The situation was different in the barrel cortex signals, whereas the radial unit size increased, the barrel signals became increasingly ambiguous and eventually disappeared (Supplementary Figure S9B). This finding highlighted the sensitivity of radial unit size in detecting fine structural details.

In summary, our approach demonstrates robustness across different species and brain regions, while also highlighting the importance of considering radial unit size when analyzing fine cytoarchitectonic structures.

## Discussion

As the brain region responsible for higher-order mental functions, the architecture profile of cortex could help us understand the generation of human intelligence and improve the artificial intelligence. But the cortical architecture is too complex to revealed only by conventional biological approaches. Multidisciplinary collaborations are necessary, such as the engineering issue for specimen imaging and computer software science for data analysis and visualization. In this study, a strategy for cortical cytoarchitectonic profile with stereo optical imaging data was introduced. This approach enabled the presentation of global cytoarchitectonic patterns on surface, while also delineating cortical laminar and regional difference in cell distribution. And some related questions and assumptions were discussed below.

### The parcellation of cortical region on surface

The surface-based data visualization of whole brain cortex was usually used in MRI-based research to present the data like the cortical thickness, the depth of sulci (Van Essen and Dierker, 2007), the myelin density and brain activity in functional task (Glasser et al., 2016). For the brains of gyrencephalic animals, the folding cortex made it less intuitive to visualize data on 2D slice images than surface, because the observers could not quickly capture the distribution pattern in a large area without the information integration from discrete images in mind. But the cytoarchitectonic study typically utilized the 2D histological slices for high-resolution optical imaging of cells. Although the slices were continuous, the axial spatial resolution was still insufficient due to the slice thickness and sampling ratio (Saleem et al., 2021) that prevented the cytoarchitectonic signal presentation on surface. In this study, the fMOST system was used for whole macaque hemisphere imaging with a stereo resolution of 0.65 μm*0.65 μm*12 μm that provided a chance to characterize the cytoarchitectonic feature in whole brain level. With this dataset, the cell distribution densities were extracted as cytoarchitectonic signals and were projected on a surface which was a representation for cortex. By this approach, the distribution pattern of cell with different size could be acquired in each cortical region and each cortical laminate, and so were the cortical laminar pattern and the cortical regional difference.

In the cytoarchitectonic feature based cortical parcellation by the traditional method on 2D sections, the multiple decisions were made for the boundary traversing the slices and the results were summarized to form the stereo parcellation. Because of the difficulty in integrating information between slices, the decisions were made independently in discrete slices and the error would be accumulated leading to mismatch between slices (Reveley et al., 2017). In contrast, when parceling the surface labeled with cytoarchitectonic signals, the observer or the algorithm took into account information in a large span and the boundary could be decided more directly with single drawing, reflecting the superiority for cortical parcellation with our method.

### The complementary information for cortical architecture

In this study, neurons were classified according to soma size, which was feasible to roughly distinguish the cell type and the existing results presented enhanced discrimination of the cortical laminates. But the cortical architecture details were far more complex than the cell size and complementary information was needed.

For an instance, the brain slices could be collected (Larsen et al., 2021) after imaging for gene expression measurement by combining single-cell sequencing with laser capture microdissection (Chen et al., 2017), the *in situ* molecule capture approach (Chen et al., 2022), the *in-situ* sequencing (Wang et al., 2018) or the *in-situ* hybridization (Fang et al., 2022). The bottleneck for integration of these method with ours was the specimen slice collection, especially for the thin slices that were difficult to maintain the integrality. Collection of the thicker slices was easier, but it also meant the lower sampling rate of imaging in current pipeline. The 3D imaging of optically cleared tissue combined with molecular detection (Pesce et al., 2022) provides an alternative approach that could offer deeper insights into neuronal characteristics and aid in elucidating their functional mechanisms. However, the alignment of individual slices is crucial and poses a significant challenge, particularly when the sample sustains damage or undergoes notable deformation during the processing phase. In our method, the specimen was securely immobilized within the hydrogel, safeguarding it from mechanical damage and minimizing distortion. Consequently, the captured images are naturally aligned, ensuring accurate and reliable results.

The nucleic acid dye propidium iodide with red fluorescence could also be combined with the green fluorescence dye for myelin (García-García et al., 2023), allowing the capture of myeloarchitectonic information at the same time, which was a crucial component of cortical architecture. The myelin signals would also assist in defining the GM/WM interface. Moreover, by recognizing the orientations of the myelin texture (Schurr and Mezer, 2021), the nerve fiber tracts could be reconstructed (Menzel et al., 2021) and contribute to the cortex-associated connection study.

Besides, the current cytoarchitectonic analysis could also be complementary with neural tracing. The regional diversity of neural connection could supplement the area with less cytoarchitectonic difference. And the cytoarchitectonic feature could serve as a reference for precise location of tracing signals in fine structures such as barrel cortex.

### The discriminative ability of this method

As it had been mentioned, the results were influenced by the size of radial unit, and the microstructure may be ignored if not already known. It was caused by the spatial resolution loss during the mean calculation of cell distribution density. Things seemed inevitable in current process. One possible solution was introducing the fourth dimension for signal smoothing by registering individuals together, and the signals would be smoothed amount the samples. This strategy had been used in mouse brain (Wang et al., 2020), but it would be challenging for cortex registration in animals like macaque and human because the high diversity between individuals (Van Essen et al., 2012). In this case, our current method could be used to produce fundamental landmarks, and the similar regions could be highlighted during the global registration. Then the local registration could be performed for microstructure detection.

Another issue was the measurement of cell size. As it was assessed based on the image binarization, it was susceptible to be affected by the staining intensity and imaging conditions. And we had found the overall signal intensity difference between samples, although cytoarchitectonic pattern was less affected. But it could still make misleading in the analysis with more variables, like interspecies or pathological comparison. Therefore, more criteria were needed to ensure equality of specimen treatment during the process.

## Conclusion

In this article, a new form of presentation for cortical cytoarchitecture was introduced. By employing the fMOST system, the whole brain hemisphere of macaque was imaged with high definition. Thus, two limitations in traditional methods were overcame. The first was the relatively low resolution of MRI-based method, which prevented the analysis at the cellular level. Depending on the high-resolution imaging data, the somas were graded by the size, and prominent laminar textures were revealed on cortical sections. Another problem was caused by the folding of cortex, that made observations from 2D sections doubtful. By establishing a cortical coordinate system, the cortex was divided into radial units and the cytoarchitectonic signals in certain depth intervals were presented on surface as tomogram. Based on these approaches, the cortical cytoarchitectures were demonstrated in both macaque and mouse and the cytoarchitectonic patterns in mouse brain were consisted with the existing finding that proved the biological reasonability of this method.

## Data availability statement

The original contributions presented in the study are included in the article/Supplementary material, further inquiries can be directed to the corresponding author/s.

## Ethics statement

The animal study was approved by Ethics Committee of the Kunming Institute of Zoology and the Kunming Primate Research Center (Approval No. IACUC18018). The study was conducted in accordance with the local legislation and institutional requirements.

## Author contributions

ZL: Data curation, Investigation, Methodology, Software, Writing – original draft. ZF: Data curation, Writing – review & editing. GL: Methodology, Writing – review & editing. AL: Data curation, Software, Visualization, Writing – review & editing. HG: Conceptualization, Funding acquisition, Project administration, Resources, Supervision, Writing – review & editing. XY: Conceptualization, Supervision, Writing – review & editing. XL: Conceptualization, Funding acquisition, Project administration, Resources, Supervision, Writing – original draft.

## Footnotes

^1^http://www.fmrib.ox.ac.uk/fsl/
