# Supplementary material for: A complementary approach for neocortical cytoarchitecture inspection with cellular resolution imaging at whole brain scale
Source: Front Neuroanat. 2024 May 23;18:1388084. doi: 10.3389/fnana.2024.1388084 (PMC11153794; doi:10.3389/fnana.2024.1388084)
Supplement: Supplementary file 1 [file Data_Sheet_1.docx]

**Supplementary Figures**


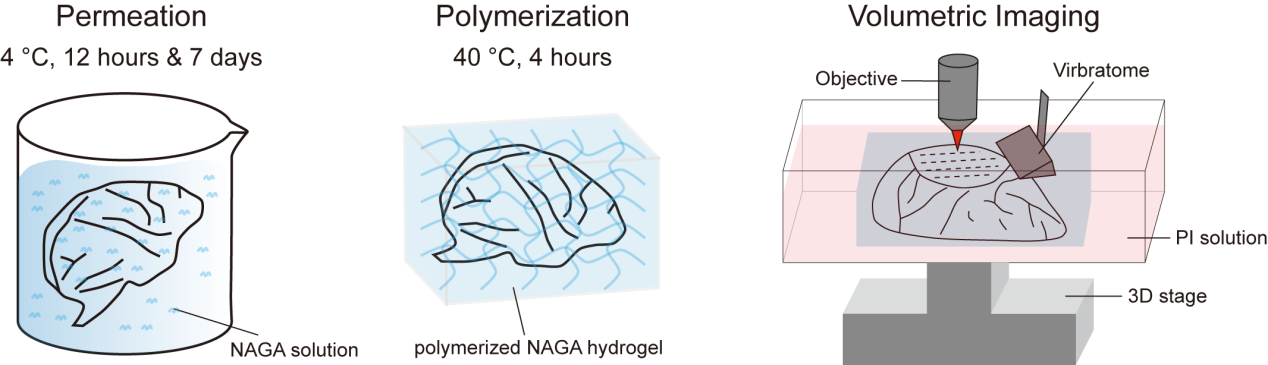


Figure S1. The schematic diagram of sample embedding and imaging.

The macaque brain was immersed in the N-acryloyl glycinamide (NAGA) solution for permeation. Then the NAGA monomers within the solution underwent polymerization through heating, resulting in the formation of the hydrogel. The embedded brain was positioned on a 3D stage and immersed in propidium iodide (PI) solution for the real-time staining of block face.


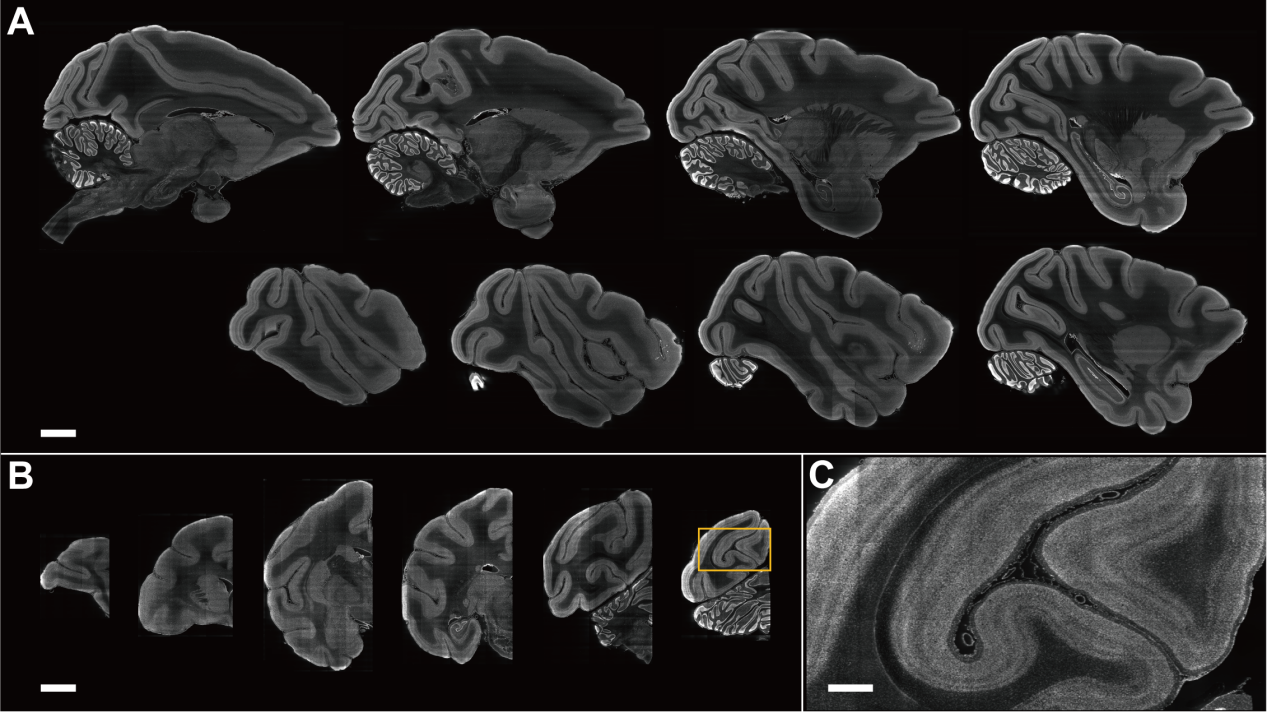


Figure S2. The 3D continuous whole macaque hemisphere imaging with high resolution.

(A-B) The representative sagittal sections and resliced coronal sections. Scale bars, 10 mm.

(C) The resliced image in the bounding box in (B) was enlarged in which the cortical structure details like layer I were still clearly presented. Scale bars, 2 mm.


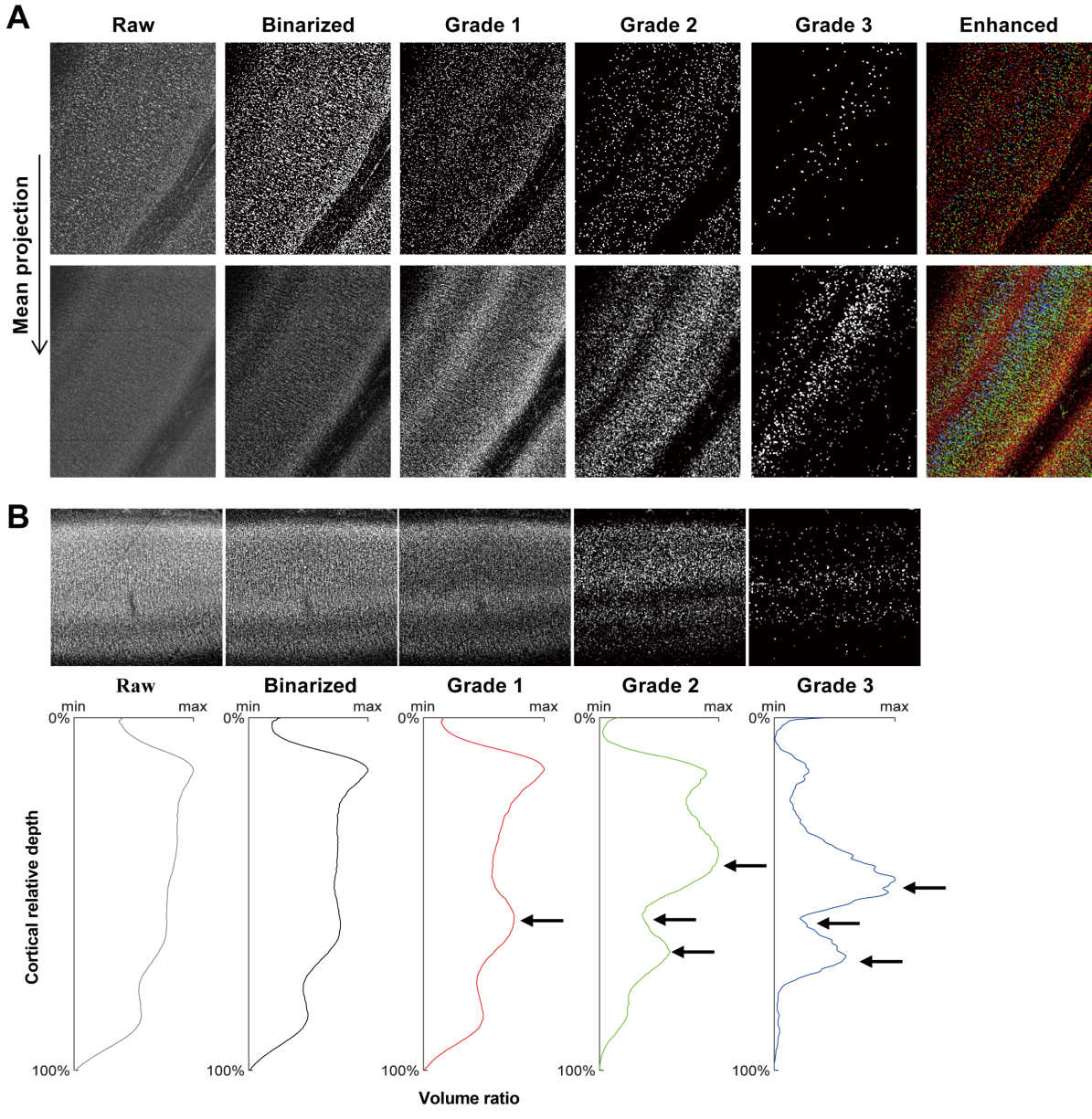


Figure S3. The enhanced images by cell grading provided better laminar contrast.

(A) The images were presented to describe how to get the enhanced image. In the first row, the raw image was binarized and the foreground were splitted into three images by cell grading and merged as cell-graded image. The images in first row were projected by the mean with adjacent images to get the images in the second row. The cortical laminates in the projections of enhanced images were more obvious than raw or the binarized-only images.

(B) The laminar contrasts were quantitatively compared. The raw, the binarized and the splited images for three grades were projected by the mean respectively in the first row. The gray values of pixels in each row were accumulated for every image and the smoothed accumulation sequences were drawn as lines below. The arrows indicated the peaks and valleys that were blurry in the lines of unenhanced images.


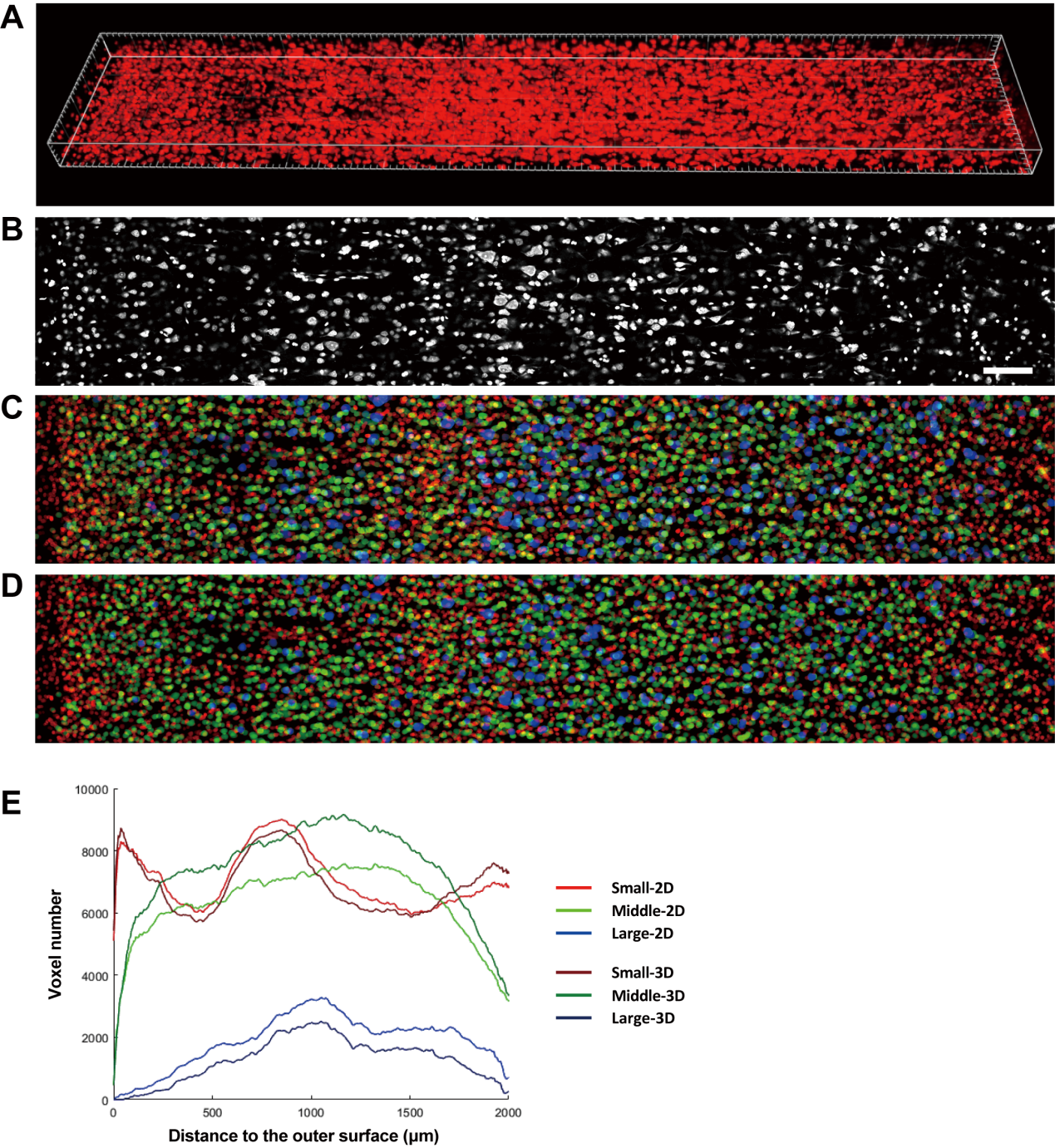


Figure S4. The comparison between 2D and 3D method in image morphological processing.

(A) The image stack presented by volume rendering.

(B) The typical section in imaging plane was shown in which the cortical laminates could be identified. Scale bars, 100 μm.

(C-D) The enhanced images corresponding to 2D and 3D morphological method presented similar laminar texture.

(E) The voxel number were accumulated for every grade in each section perpendicular to the cortex radial direction. And the accumulated voxel number series were drawn as curves.


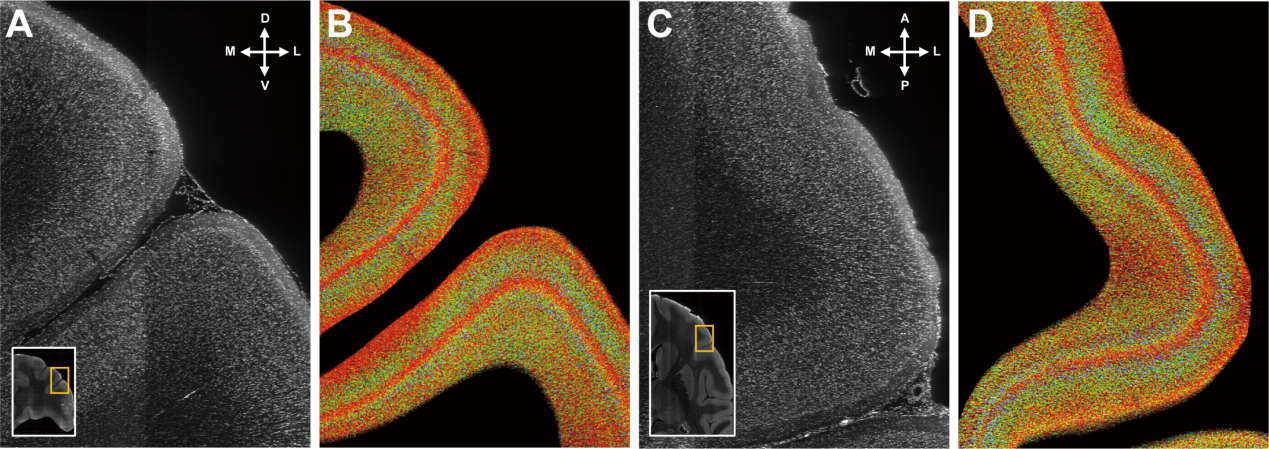


Figure S5. The limited effect of section angle on cytoarchitectonic signal pattern.

(A, C) The parts of coronal and horizontal section were selected in which the cortex was curled relative to the sagittal plane of imaging.

(B, D) The cortical laminar textures in the enhanced images were still coherent along the cortex.


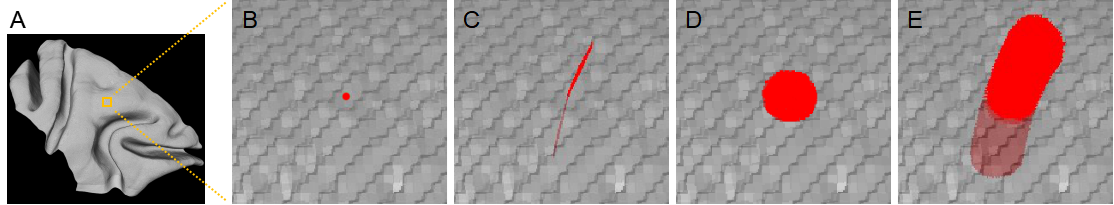


Figure S6. The schematic diagram of radial unit.

(A) The mid-thickness surface on which the position of B-E was indicated with a box.

(B) The point labeled a voxel on the surface which was the center of a radial unit.

(C) The fascicular pattern indicated all points that projected to the voxel in B

(D) The disk-shape pattern indicated the voxels that located on the surface and were adjacent to the voxel in B

(E) The columnar pattern indicated all points that projected to the voxel in D. And these points formed a radial unit


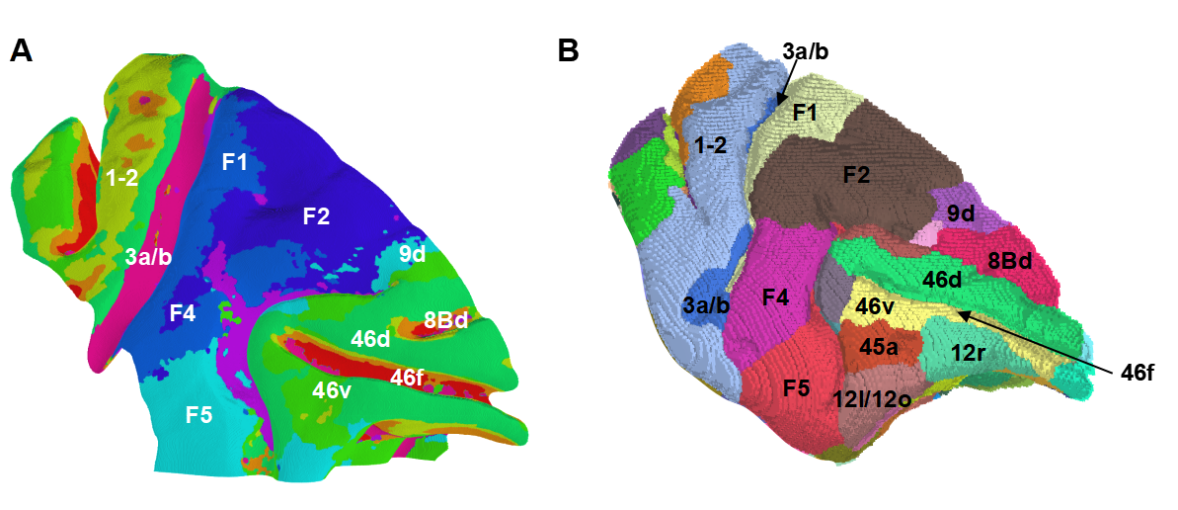


Figure S7. The comparison of cortical parcellation between ours and the conducted one.

(A) The cytoarchitecture-based parcellation on surface in our study.

(B) The partial volume rendering of the macaque brain atlas

The text labeled the abbreviation of region name.


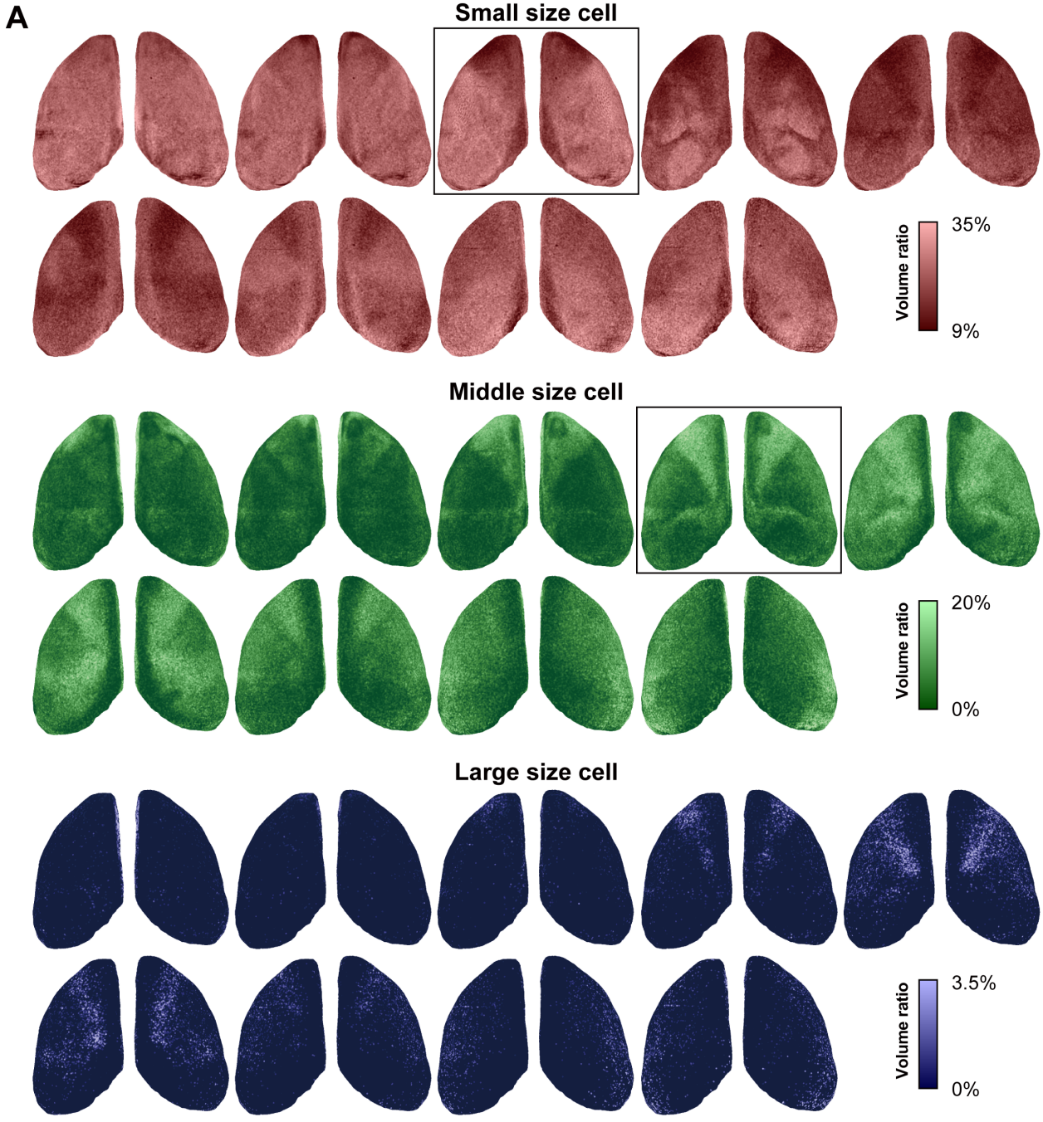

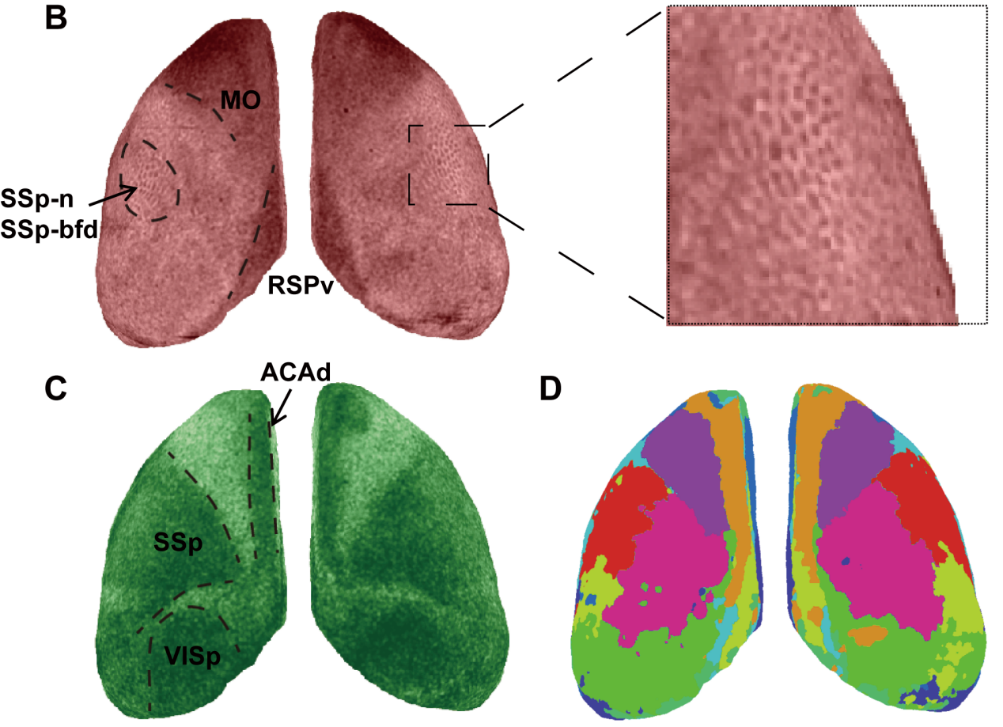


Figure S8. The methodological verification for biological reasonability using mouse brain.

(A) The tomographic presentation of cytoarchitectonic signal in mouse brain. And the subgraphs displayed in (B-C) were labeled with boxes.

(B-C) The typical tomographic presentation from mouse brain were selected and the regional boundaries were labeled with dotted line. The barrel field of primary somatosensory area was enlarged. The abbreviation of the region name: MO, somatomotor areas; SSp, SSp-n and SSp-bfd, primary somatosensory area and the subregions for nose and barrel field; RSPv, the ventral part of retrosplenial area; ACAd, the dorsal part of anterior cingulate area; VISp, primary visual area.

(D) The cortex was parceled by clustering and the covering regions were painted with colors.


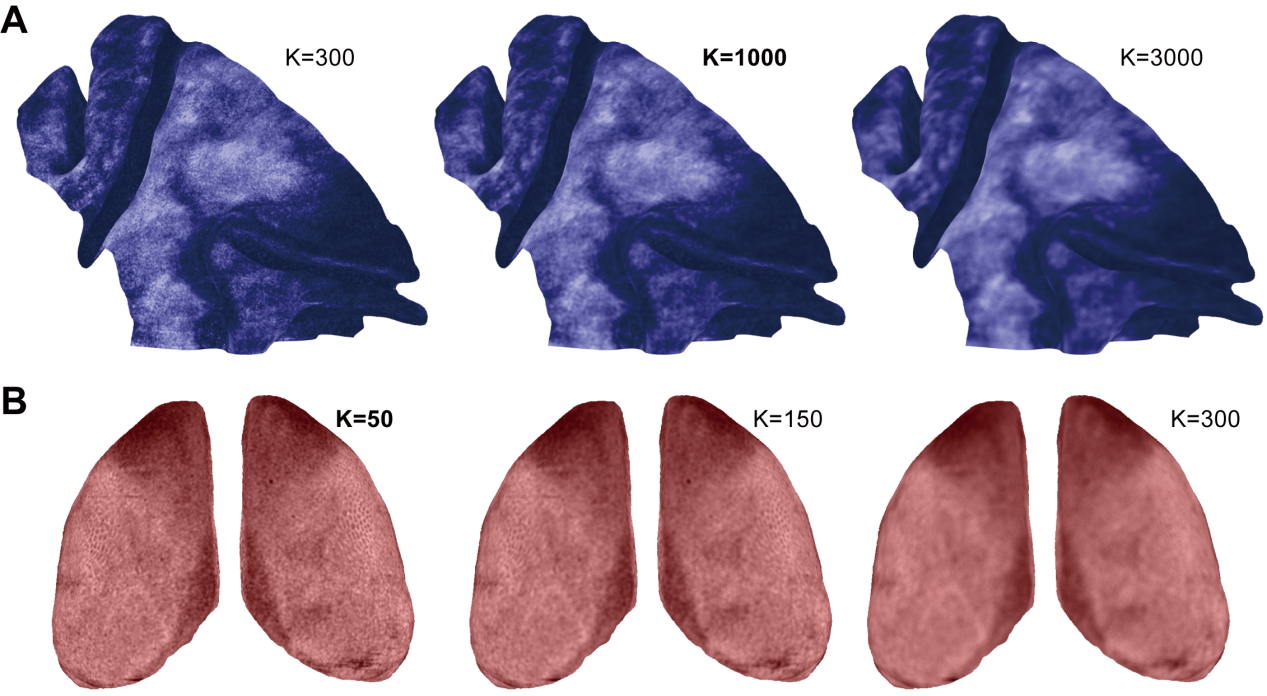


Figure S9. The cortical radial unit size assessment.

(A) The third surface in Figure 4C was chosen as a representative for its relatively rich texture details. The K-value was the parameter used in K-Nearest Neighbor algorithm to control the radial unit size, and it was changed for comparison.

(B) The similar comparation was done to assess the effect on barrel cortex in mouse. The K-values with bold font were used in main text.
